# *Pax3* and *Pax7* Exhibit Distinct and Overlapping Functions in Marking Muscle Satellite Cells and Muscle Repair in a Marine Teleost, *Sebastes schlegelii*

**DOI:** 10.3390/ijms22073769

**Published:** 2021-04-05

**Authors:** Mengya Wang, Weihao Song, Chaofan Jin, Kejia Huang, Qianwen Yu, Jie Qi, Quanqi Zhang, Yan He

**Affiliations:** 1 MOE Key Laboratory of Molecular Genetics and Breeding, College of Marine Life Sciences, Ocean University of China, Qingdao 266003, China; mengya0828@163.com (M.W.); songweihao8@163.com (W.S.); 18663360681@163.com (C.J.); 15071255840@163.com (K.H.); Carolineyqw@163.com (Q.Y.); qijie@ouc.edu.cn (J.Q.); qzhang@ouc.edu.cn (Q.Z.); 2 Laboratory of Tropical Marine Germplasm Resources and Breeding Engineering, Sanya Oceanographic Institution, Ocean University of China, Sanya 572000, China

**Keywords:** *Pax3*, *Pax7*, muscle satellite cell, muscle repair, *Sebastes schlegelii*

## Abstract

*Pax3* and *Pax7* are members of the *Pax* gene family which are essential for embryo and organ development. Both genes have been proved to be markers of muscle satellite cells and play key roles in the process of muscle growth and repair. Here, we identified two *Pax3* genes (*SsPax3a* and *SsPax3b*) and two *Pax7* genes (*SsPax7a* and *SsPax7b*) in a marine teleost, black rockfish (*Sebastes schlegelii*). Our results showed *SsPax3* and *SsPax7* marked distinct populations of muscle satellite cells, which originated from the multi-cell stage and somite stage, respectively. In addition, we constructed a muscle injury model to explore the function of these four genes during muscle repair. Hematoxylin–eosin (H–E) of injured muscle sections showed new-formed myofibers occurred at 16 days post-injury (dpi). ISH (in situ hybridization) analysis demonstrated that the expression level of *SsPax3a* and two *SsPax7* genes increased gradually during 0–16 dpi and peaked at 16 dpi. Interestingly, *SsPax3b* showed no significant differences during the injury repair process, indicating that the satellite cells labeled by *SsPax3b* were not involved in muscle repair. These results imply that the muscle stem cell populations in teleosts are more complicated than in mammals. This lays the foundation for future studies on the molecular mechanism of indeterminant growth and muscle repair of large fish species.

## 1. Introduction

Skeletal muscle, as the main edible part of fish, accounts for 40–60% of the entire body weight [1]. The muscle growth rate of fish determines the benefits of aquaculture. Unlike mammalian postnatal muscle growth patterns that only increase the size of existing muscle fibers [2], most fish muscles exhibit a growth pattern of both hyperplasia and hypertrophy [3]. Muscle satellite cells, also named muscle stem cells, have the ability to self-renew and differentiate, providing a source of new muscle fibers during muscle growth and repair [4]. In adult mammals, muscle satellite cells are in a resting state, and they begin to proliferate and differentiate for muscle damage repair only after muscle injury. However, the proliferation and differentiation of muscle satellite cells have always been active in adult fish, especially in large economic fish species which show indeterminate growth [5]. This implies that large fish have different regulation mechanism of muscle satellite activation from mammals, but the mechanism is still unclear.

Muscle satellite cells were first observed in the skeletal muscles of frogs by electron microscopy and were “wedged” between the plasma and basement membranes of muscle fibers in 1961 [6]. For a long time following its discovery, electron microscopy was the only reliable method of identification. More recently, the discovery of a few molecular markers expressed by satellite cells made them more accessible for study at the molecular level. *Pax3* and its paralog, *Pax7*, were proved to be molecular markers of muscle satellite cells in many different species [7,8,9,10,11]. These two genes both belong to the *Pax* gene family that encodes highly conserved transcription factors and plays key roles in tissue and organ formation during development [12,13,14]. *Pax3* is necessary for maintaining the migration of these cells in the somite and their migration to the myogenic sites [15]. High levels of *Pax3* could interfere with the differentiation of muscle cells in embryos and adults [16,17], and it also plays crucial roles in adult muscle homeostasis and skeletal muscle repair [18,19], which is enough to induce the expression of *MyoD* and *Myf5* by direct binding and trans-activation of their enhancers in vitro [20,21]. Recent research has shown that the expression of *Pax3* can promote the contribution of muscle satellite cells to the balance of muscle structure and function [22] and make muscle satellite cells display a functional heterogeneity in responding to environmental stress [23]. *Pax7* is the first specific marker factor identified in a muscle satellite cell in a resting and activated state, which is crucial for the proliferation and differentiation of muscle satellite cells, as well as adult skeletal muscle development and repair [7,24]. The muscle size of *Pax7*^−/−^ mice was significantly reduced, the number of nuclei of muscle fibers was about 50% of the normal nuclei, and the diameter of muscle fibers was also significantly reduced. Moreover, the mice showed poor vitality and usually died within the first three weeks after birth [25,26]. Mechanical injury to adult zebrafish skeletal muscle lead to an increase in the number of *Pax7* positive cells in the injured area, and the muscle repair function of zebrafish with *Pax7a* and *Pax7b* mutations was impaired [27].

Despite decades of effort towards tracing the embryonic origin of muscle satellite cells, the answer is still ambiguous in different species. Studies have shown that most skeletal muscle derived from the somite is formed by epithelial spheres of the mesoderm of the gastrointestinal embryo [16,28,29]. As the somite matures, the back part of the somite develops into the dermomyotome. A key step in the growth and development of skeletal muscle is the formation of the dermomyotome, and the muscle satellites cells originate from sarcomere cells and further develop into myoblast cells [30]. Then, the myoblast cells fuse with each other and develop into multinucleated myotubes, which fuse to form muscle fibers. Experiments using bird models for interspecies transplantation, as well as the conclusions of Randall’s study on *Xenopus laevis*, both support this view [30,31]. However, studies of zebrafish and mouse embryos have proven that muscle satellite cells are derived from somatic frontal cells and embryonic dorsal aorta, respectively [32,33]. The questions of when muscle satellite cells originate during embryogenesis and how they are involved in the muscle repair have still not been answered in fish species with an indeterminate growth pattern.

Black rockfish (*Sebastes schlegelii*) is an economically important marine species in China and exhibits an indeterminate muscle growth pattern [34]. In order to study the functions of muscle stem cells in muscle growth and repair, we characterized *Pax3* and *Pax7* genes in *S. schlegelii* and explored their function in marking muscle satellite cells and muscle repair. We found that *Pax3* and *Pax7* genes label distinct muscle stem cell populations in *S. schlegelii*. Our results provide evidence that the embryonic origin of muscle satellite cells varies between teleosts and mammals. This lays the foundation for the future studies on the molecular mechanism of indeterminate growth and muscle repair of large fish species.

## 2. Results

### 2.1. Gene Structure, Synteny Analyses, and Evolution of SsPax3 and SsPax7

**Figure 1:**
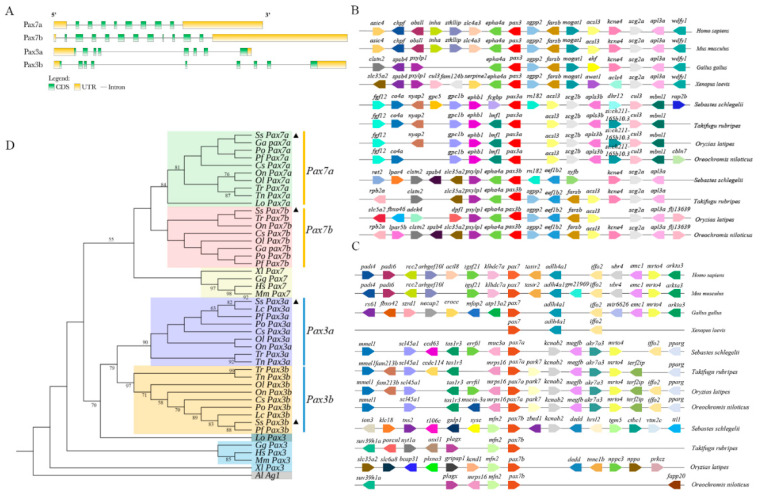
Characterization of *SsPax3* and *SsPax7* in *Sebastes schlegelii*. (**A**) Gene structures of *SsPax3* and *SsPax7*. Exons are shown in the green box, untranslated regions (UTR) are shown in the yellow box, and introns are shown in the straight line. (**B**) Syntenic analysis of *Pax3* genes on the chromosome or scaffold. (**C**) Syntenic analysis of *Pax7* genes on the chromosome or scaffold. Different colored pentagons represent different genes, and the direction of pentagons corresponds to the direction of genes in the chromosome or scaffold. (**D**) Phylogenetic analysis of *Pax3* genes and *Pax7* genes in tetrapods and teleosts. Supported bootstrap values are listed at the tree nodes. The species and accession numbers are shown in Table S1. The black triangles mark the *Pax3* genes and *Pax7* genes of *Sebastes schlegelii*.

Based on the transcriptome and genome database of *S. schlegelii*, we identified the exon–intron structure of *Pax3* and *Pax7* by hand (Figure 1A). *SsPax3* and *SsPax7* genes displayed differences in gene structure. *SsPax3a* contained eight exons and seven introns, while *SsPax3b* contained nine exons and eight introns. By contrast, *SsPax7a* contained eight exons and nine introns, while *SsPax7b* contained 11 exons and 12 introns. The syntenic map confirmed the speculation that the teleost *Pax3* and *Pax7* paralogs originated from the unique whole genome duplication (WGD) of teleost instead of fragment duplication. As shown in Figure 1B, the *Pax3* genes and neighboring genes were conserved among investigated teleost species. Furthermore, the adjacent genes of *Pax3b* in teleosts were more conserved than *Pax3a*. The genes near *SsPax3a* were different from the genes near *SsPax3b* (Figure 1B). Compared with *Pax7b* in different teleost fishes, the adjacent genes of *Pax7a* were more conservative, although some gene loss and mutation were observed (Figure 1C). The phylogenetic analysis showed that the *SsPax3a* and *SsPax3b* of *S. schlegelii* were divided into two distinct evolutionary branches, and put into the evolutionary branches of *Pax3a* and *Pax3b* of teleosts, respectively (Figure 1D). The two *SsPax7* genes also showed the same evolutionary relationship, indicating that *SsPax3* and *SsPax7* were homologous to the mammalian *Pax3* and *Pax7* genes, respectively. The results provide further evidence that additional gene replication events had occurred in the teleost fish.

### 2.2. Sequence Analysis and 3D Protein Structures of SsPax3 and SsPax7

**Figure 2:**
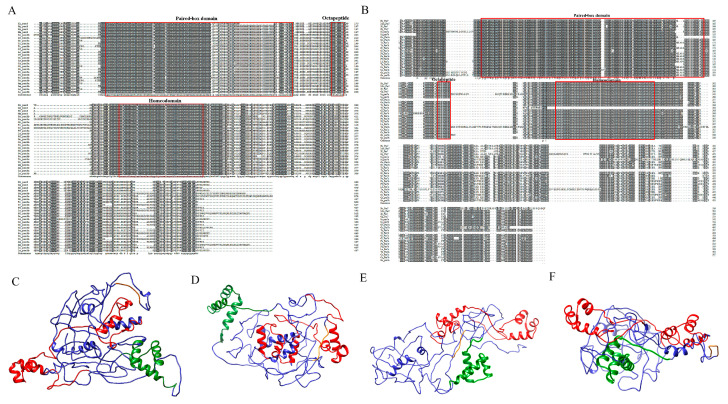
Sequence analysis and 3D protein structures of *SsPax3* and *SsPax7*. Multiple alignment of the deduced amino acid sequences of *Pax3* genes (**A**) and *Pax7* genes (**B**) from teleosts and tetrapods. The amino acid sequences of teleost *Pax3* genes and *Pax7* genes contained a paired-box domain, a homeodomain, and an octapeptide which were boxed. The dark-gray shadow shows the same amino acid residues, and the light-gray areas represent the identical amino acid residues. The species names and accession numbers are available in Table S1. (**C**–**F**) Predicted three-dimensional structure of two *SsPax3* genes and two *SsPax7* genes. Paired-box domain is shown in red, homeodomain is shown in green, and octapeptide is shown in orange.

Multiple alignment results showed that the amino acid sequences of *SsPax3* and *SsPax7* were highly conserved, and both of them contained a paired-box domain (PD), an octapeptide motif (OP), and a homeodomain (HD) with specific DNA binding properties. However, there was a nonconservative region in both of these genes at the C-terminal, indicating that some differentiation may occur in their functions (Figure 2A,B). We constructed the *SsPax3* and *SsPax7* 3D protein structures by Phyre2 and Chimera (http://www.sbg.bio.ic.ac.uk/phyre2/html/page.cgi?id = index, accessed on 11 January 2021). As shown in Figure 2C–F, each *Pax* protein contained a paired-box domain (red color), octapeptide (orange color), and homeodomain (green color), and the paired-box domain contained two α-helices, while homeodomain contained three α-helices.

### 2.3. Expression of S. schlegelii Pax3 and Pax7 in Different Tissues

**Figure 3:**
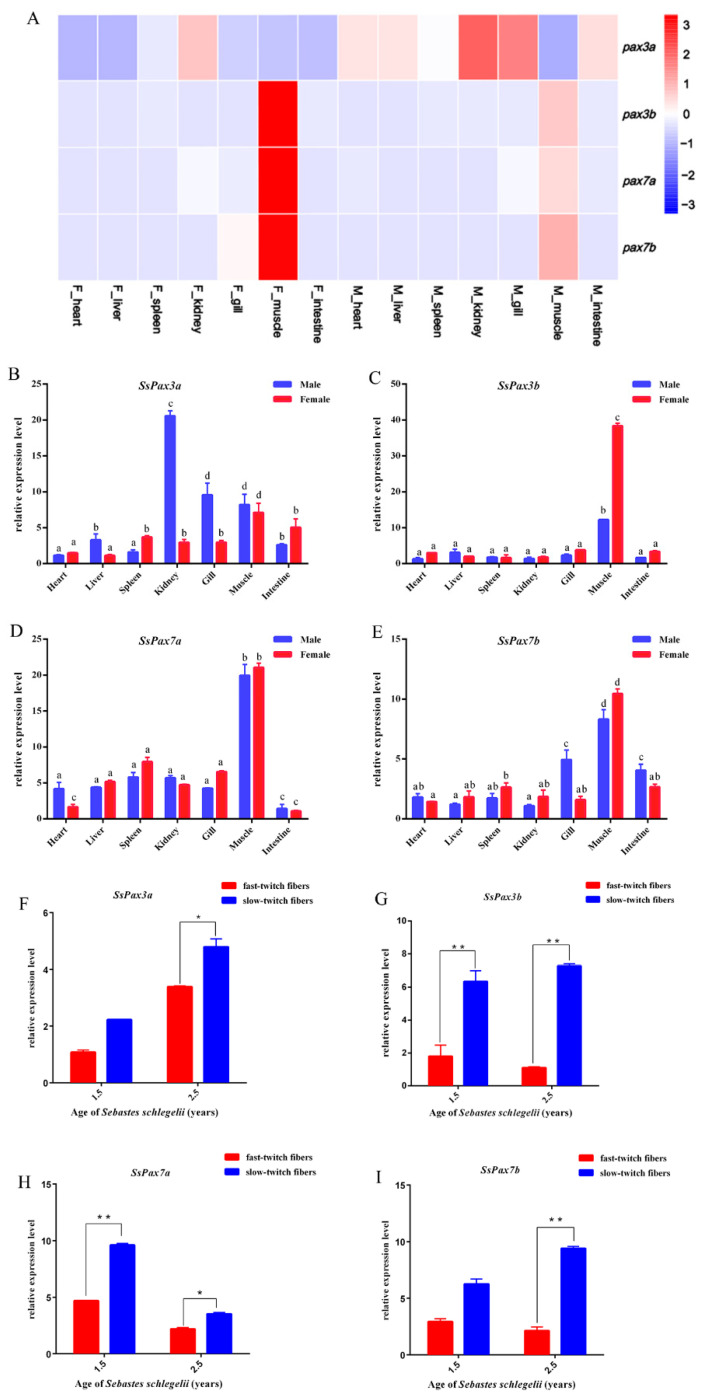
Expression analysis of *S. schlegelii Pax3* and *Pax7* genes in different tissues. (**A**) Heat maps show gene expression patterns of *S. schlegelii Pax3* and *Pax7* transcripts based on TPM value, with blue and red indicating low and high expression levels, respectively. Each column represents an independent tissue or embryo sample, and each row represents one gene. (**B**–**E**) qRT-PCR analysis of *SsPax3* and *SsPax7* in different tissues; different letters indicate statistical significance (*p* < 0.05). (**F**–**I**) qRT-PCR analysis of *SsPax3* and *SsPax7* in fast-twitch fibers and slow-twitch fibers in different ages of *S. schlegeli.* Vertical bars represent the mean ± SEM (n = 3). * indicates statistical significance (*p* < 0.05), ** indicates statistical significance (*p* < 0.01).

We obtained the temporal expression patterns of *SsPax3* and *SsPax7* in different tissues based on *S. schlegelii* transcriptome data and then validated by qRT-PCR. As shown in Figure 3A–E, the expression patterns of *SsPax3a* and *SsPax3b* in tissues were distinct. Compared with *SsPax3a*, the expression level of *SsPax3b* was higher in muscle. However, the expression of two *SsPax7* genes had similar expression patterns in different tissues, with high expression levels in muscle and relatively low expression levels in other tissues. Interestingly, the expression levels of these genes in female muscles were always higher than in male muscles. The expression levels of these four genes were further validated by qRT-PCR, which was consistent with the transcriptome data. Moreover, we analyzed the expression of *SsPax3* and *SsPax7* in fast-twitch fibers and slow-twitch fibers by qRT-PCR. Four genes were all expressed at a higher level in the slow-twitch fibers than in the fast-twitch fibers at different ages of *S. schlegelii* (Figure 3F–I). This is consistent with the previous finding that the number of satellite cells in slow-twitch fibers is 5–6 times more than that in fast-twitch fibers [35]. 

### 2.4. Muscle Satellite Cells Identification in S. schlegelii

**Figure 4:**
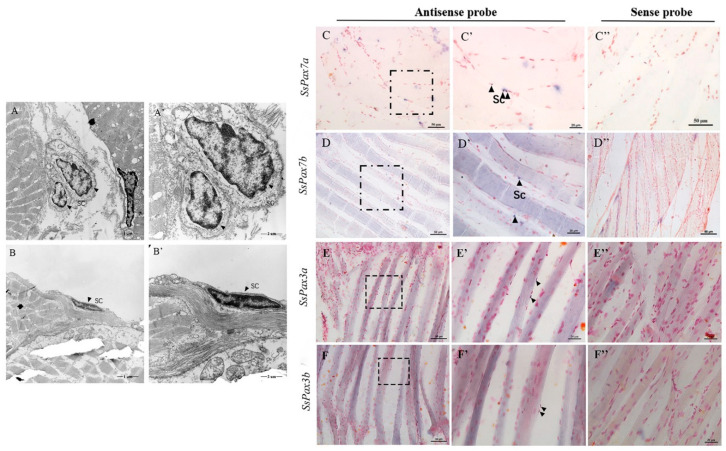
Muscle satellite cell identification by TEM and ISH of *SsPax3* and *SsPax7* in *S. schlegelii*. TEM microphotographs of satellite cells (black arrowheads) from slow-twitch muscle fiber domains of *S. schlegelii* (**A**,**A’**,**B**,**B’**). In situ hybridization was conducted with DIG-labeled *SsPax7a* (**C**,**C’**), *SsPax7b* (**D**,**D’**), *SsPax3a* (**E**,**E’**), and *SsPax3b* (**F**,**F’**) antisense probes, or sense probes as a negative control (**C’’**–**F’’**). The positive signals were observed in satellite cells (black arrowheads) at the edge of slow-twitch muscle fibers.

Satellite cells were observed by transmission electron microscopy (TEM) in mammalian muscles [25]. We used TEM to determine the presence of satellite cells in *S. schlegelii* skeletal muscle, and cells with irregular shape and dense nuclear heterochromatin were found at the edge of slow muscle fibers (Figure 4A,A’,B,B’). Previous studies have shown that *Pax7*-positive cells are located external to the muscle membrane and underneath the basal lamina of the surrounding basement membrane. In our study, we also found that significant hybridization signals of *SsPax3* and *SsPax7* genes all appeared at the edge of slow-twitch muscle fibers of *S. schlegelii* (Figure 4C,C’,D,D’,E,E’,F,F’).

### 2.5. Tracking the Embryonic Origin of Muscle Satellite Cells in S. schlegelii

**Figure 5:**
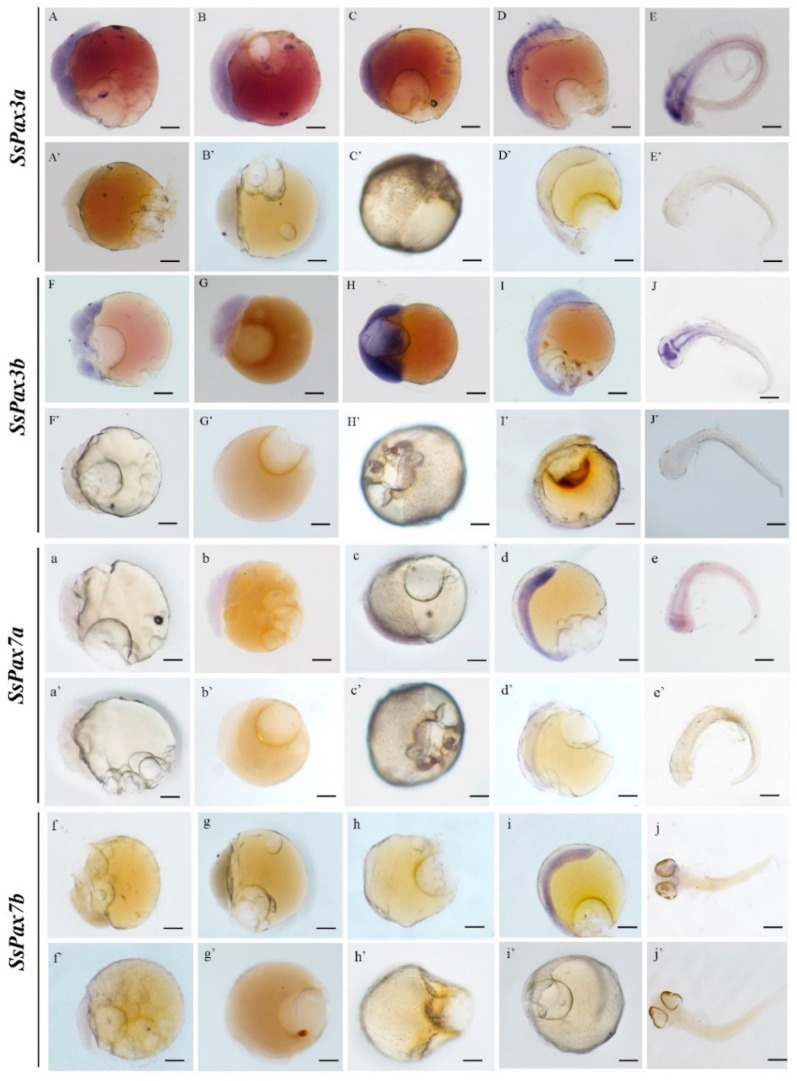
The expression patterns of *SsPax3* and *SsPax7* transcripts at different embryonic developmental stages of *S. schlegelii*: multi-cells stage (**A**,**a**,**F**,**f**), blastula (**B**,**b**,**G**,**g**), gastrula (**C**,**c**,**H**,**h**), somite (**D**,**d**,**I**,**i**), and pre-hatching (**E**,**e**,**J**,**j**). WISH was performed with DIG-labeled *SsPax3a* (**A**–**E**), *SsPax3b* (**F**–**J**), *SsPax7a* (**a**–**e**), and *SsPax7b* (**f**–**j**) antisense probes, or sense probes as a negative control (**A’**–**J’**,**a’**–**j’**). Positive signals were stained with blue, whereas the negative controls weren’t stained. Scale bars = 100 μm.

To track the embryonic origin of muscle satellite cells, we detected the mRNA signals of *SsPax3* and *SsPax7* of *S. schlegelii* at different embryonic developmental stages. In the early stage of embryo development, the signal distributions of *SsPax3a* and *SsPax3b* were similar and both first appeared in all blastomeres at the multi-cell stage, blastula stage, and gastrula stage (Figure 5A–C,A’–C’). As shown in Figure 5D, the signals of *SsPax3a* were scattered in the somites at the somite stage, while they appeared in the brain and lateral neural plate at the pre-hatching stage (Figure 5E). The expression patterns of two *SsPax3* genes had a slight difference at the somite stage, whereby the mRNA signals of *SsPax3b* were more obvious than *SsPax3a* in the lateral neural plate (Figure 5E’). Conversely, the signals of two *SsPax7* genes were initially detected at the somite stage; *SsPax7a* had a similar expression to *SsPax3b* at the somite stage and pre-hatching stage, but *SsPax7b* was expressed in the whole body at somite stage (Figure 5i) and concentrated in the brain region during the early stage of hatching (Figure 5j). No signal was observed by sense probe of the four genes at any stage (Figure 5a–j,a’–j’).

### 2.6. Histological Examination of S. schlegelii Muscle after Injury

**Figure 6:**
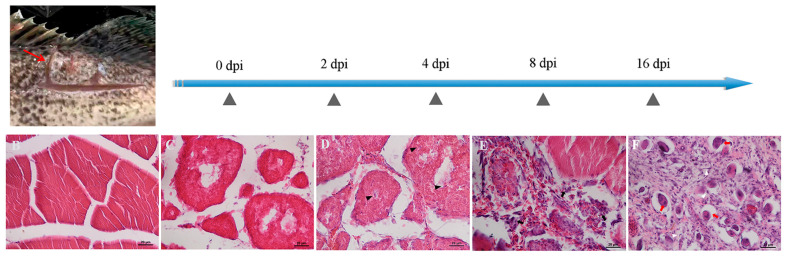
Hematoxylin–eosin staining was conducted on the injured sections of muscle at different time points after injury. (**A**) The site of the wound (red arrow). (**B**) The muscle at 0 dpi. (**C**) The architecture of the muscle was destroyed at 2 dpi. (**D**) PMNs (arrowheads) were observed in the injured area at 4 dpi. (**E**) Round-shaped MNCs (arrows) were observed in the injured area at 8 dpi. (**F**) FBCs (white arrowheads) were observed in the injured area at 16 dpi, followed by regenerated multinucleated myotubes (red arrows). Scale bar = 20 μm.

We made sections of the muscle tissue at the site of the injury, stained these with hematoxylin–eosin (H–E), and observed hemorrhage, edema, and degeneration appearing in the injured region of skeletal muscles (Figure 6). As the results show, the structure of myofibers was intact at 0 dpi (Figure 6B). We observed rapid necrosis of myofibers and the activation of an inflammatory response, further leading to the destruction of muscle architecture within 2–4 dpi, and a small number of poly-morphonuclear cells (PMNs) appeared in the injured areas (Figure 6C,D). Until 8 dpi, there were a large number of PMNs, and round mononuclear cells (MNCs) appeared in wound areas (Figure 6E). At 16 dpi, a mass of spindle-shaped fibroblastic cells (FBCs) appeared in the injured regions, concomitant with regenerated multinucleated myotubes (Figure 6F).

### 2.7. The Function of SsPax3 and SsPax7 in Injured Muscle during Repair

**Figure 7:**
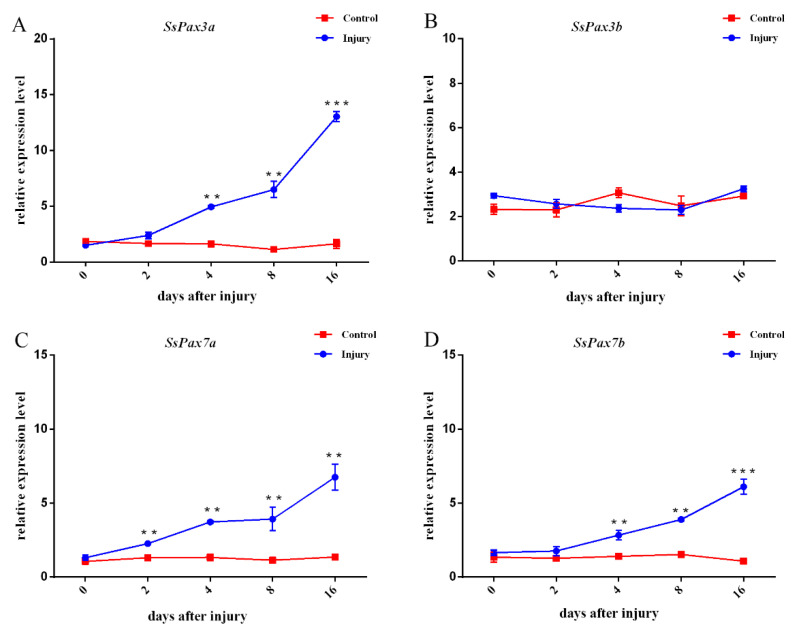
The relative expression levels of *SsPax3* (**A**,**B**) and *SsPax7* (**C**,**D**) genes at different time points after injury. Vertical bars represent the mean ± SEM (*n* = 3). ** indicates statistical significance (*p* < 0.01); *** indicates statistical significance (*p* < 0.001).

**Figure 8:**
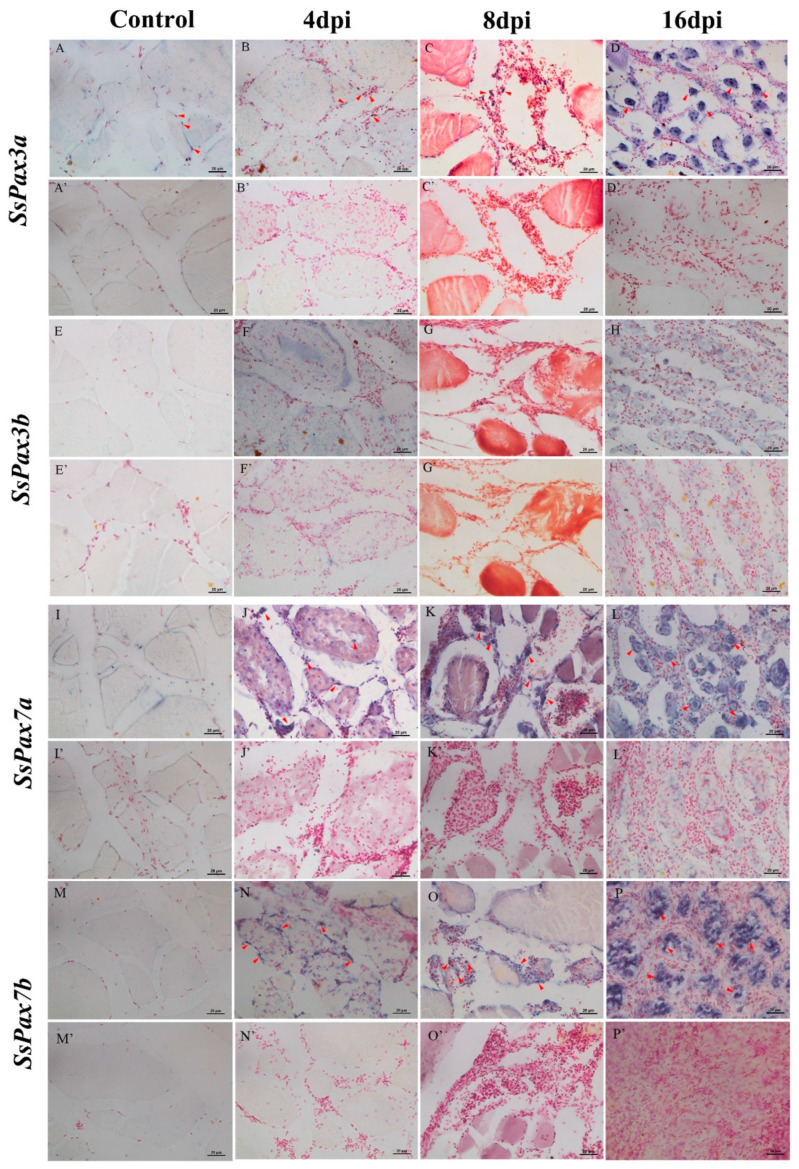
The expression patterns of *SsPax3* and *SsPax7* transcripts in the injured sections of muscle during 0–16 dpi. ISH was conducted with DIG-labeled *SsPax3a* (**A**–**D**), *SsPax3b* (**E**–**H**), *SsPax7a* (**I**–**L**), and *SsPax7b* (**M**–**P**) anti-sense probes or sense probes as negative control (**A’**–**P’**). The positive signals were observed in poly-morphonuclear cells, new-formed round monocytes and multinucleated myotubes (red arrowheads), whereas the negative control showed no obvious signals. Scale bar = 20 μm.

The expression patterns of SsPax3 and SsPax7 genes were investigated during the regeneration process after injury (Figure 7). *Pax3a* and *Pax3b* showed a significantly different response to muscle injury. As shown in Figure 7A, the expression level of *SsPax3a* increased gradually and reached the highest expression level at 16 dpi. However, during 0–16 dpi, the expression of *SsPax3b* showed no significant change compared with the control (Figure 7B). Moreover, the expression levels of *SsPax7a* and *SsPax7b* were significantly elevated over the generation process (Figure 7C,D). ISH was applied to further explore the role of *SsPax3* and *SsPax7* in the process of muscle repair. Consistently, the ISH results of *SsPax3a* showed an up-regulation trend during muscle repair in the anti-sense groups (Figure 8B–D). At 4 dpi, few signals were found in the poly-morphonulcear cells (PMNs) (Figure 8B), but more signals appeared in the PMNs and the new-formed round monocytes at 8 dpi (Figure 8C). Until 16 days post-injury, strong signals were found in spindle-shaped fibroblastic cells and multinucleated myotubes (Figure 8D), and no signal was detected in the sense probe groups (Figure 8A’–D’). By contrast, no significant signal of *SsPax3b* was observed during the repair of injured muscles in both the anti-sense groups (Figure 8E–H) and sense groups (Figure 8E’–H’). The number of *SsPax7*-expressing cells were higher in the area of muscle injury at 4 dpi compared with *SsPax3*, and the mRNA hybridization signals of two *SsPax7* genes appeared not only at the edges of muscle fibers, but also at the damaged area of muscle fibers (Figure 8J,N). Similar to the expression of *SsPax3a* in the injured muscle region, the strong signals of *SsPax7a* (Figure 8K) and *SsPax7b* (Figure 8O) also appeared in the PMNs and the new-formed round monocytes at 8 dpi and appeared in the multinucleated myotubes at 16 dpi (Figure 8L,P).

## 3. Discussion

In our study, we identified two homologous *Pax3* genes and two homologous *Pax7* genes in *S. schlegelii*. As a previous study has shown, *Pax3* and *Pax7* are different genes in tetrapods, while there is only a single ancestral gene *Pax3/7* in amphioxi [36]. Due to the presence of additional fish-specific genome duplication (FSGD) in the lineage of teleosts, there are four duplicates, *Pax3a*/*b* and *Pax7a*/*b,* such as in olive flounders (*Paralichthys olivaceus*) and fugu (*Takifugu rubripes*) [37,38,39]. Our findings of two *SsPax3* genes and two *SsPax7* genes in *S. schlegelii* also supports the additional FSGD theory. Previous studies have reported that the *Pax3* and *Pax7* genes of mammals are closely related in structure and function. Based on the protein structures of *SsPax3* and *SsPax7*, the paired-box domain showed greater differences in structure among four genes compared to the homeodomain. As described in previous studies, the helices region of the paired-box domain may contact DNA sequences and determine the specificity of DNA binding [40]. The divergence of the paired-box domain in four proteins may suggest that they bind to distinct DNA sequences.

In previous studies, *Pax3* genes and *Pax7* genes shared common expression in mouse embryos [41,42,43], while two *Pax3* genes and two *Pax7* genes showed distinct expression during embryogenesis in some teleosts [37,39,44]. Some scholars have confirmed through the study of zebrafish embryos and mouse embryos that muscle satellite cells are derived from cells at the leading edge of the somite and the dorsal aorta of the embryo, respectively [18,32,45,46]. It has also been reported that the mRNA signals of *Pax3* are initially observed at the neural plate at the gastrula stage in embryos in *Xenopus laevis* [41,47]. In olive flounders, *Pax3a* signals were detected in the somites while the mRNA transcripts of *Pax3b* were only expressed in the new-forming somites [37].The duplicated *Pax7* genes also showed distinct expression patterns during embryogenesis in olive flounders [39]. Interestingly, in this study, the expression level of *SsPax3* genes at different embryonic stages were distinct with the results in the olive flounders and zebrafish homologues. The signals of *Pax3a* and *Pax3b* of *S. schlegelii* were detected from the multi-cells stage and lasted until the hatching stage. Conversely, the expression of two *SsPax7* first appeared in the somite stage in *S. schlegelii*. A previous study has proven that both the transcripts of *Pax7a* and *Pax7b* were initially detected in the somite stages, but the expression regions of them were slightly different [39]. Of note, the initial stage used to detect the expression of *Pax3/7* in olive flounders was the neural stage; therefore, there is no further information on whether *Pax3/7* express in stages earlier than the neural stage. Taken together, our results indicate that *SsPax3* and *SsPax7* mark different populations of muscle stem cells in *S. schlegelii*. In addition, the muscle satellite cells labeled by *SsPax3* originated earlier than the somite stage, which conflicted with the previous report that muscle satellite cells originated from mesoderm cells during the somitogenesis phase [29,48]. To our knowledge, this is the first report in teleost fish that traced the earliest embryonic origin of muscle satellite cells. This also indicated the heterozygosity of muscle satellite cells in teleosts, implying their complexity. According to previous reports, skeletal muscle growth and repair after birth depends on satellite cells characterized by *Pax7* expression [4,27,49]. Inflammatory cells and myogenic cells appeared at the site of injured muscles, and were usually accompanied by activation of mononucleated cells [50]. Recent studies have shown that injured muscles release factors to activate inflammatory cells In response to muscle injury, quiescent satellite cells were activated, migrated to the site of injury, and then differentiated into new fibers to repair and replace the damaged myofibers [51,52]. In our study, a large number of round-shaped mononuclear cells (MNCs) appeared in wound areas at 8 dpi of *S. schlegelii*, suggesting satellite cells were activated to proliferate and differentiate. After that, the MNCs fused to necrotic fibers for repair or fused to each other to form myotubes for the formation of new fibers (Figure 5); this rapid and dynamic process involved the activation of complicated cellular responses, and it was enough to prove that *S. schlegelii* can stimulate their own muscle repair function after injury. Previous studies have confirmed that *Pax3* and *Pax7* both have overlapping and different functions in mouse regenerative myogenic satellite cells. Here, qRT-PCR analysis of *SsPax3/7* genes showed that the expression of *SsPax3a* and two *SsPax7* genes was up-regulated during muscle repair, but *SsPax3b* did not show response to muscle injury. Accordingly, the ISH of *SsPax3/7* genes in regenerating muscle showed that there were strong signals of *SsPax3a* and two *SsPax7* genes in regenerative myotubes and new myofibers, providing further evidence for the viewpoint that *SsPax3a* and *SsPax7* might have an important impact on repairing damaged muscle, while *SsPax3b* might play a role in the self-renewal of satellite cells and muscle growth.

## 4. Materials and Methods

### 4.1. Ethics Statement

This study was approved by the College of Marine Life Sciences, Ocean University of China Institutional Animal Care and Use Committee on 10 October 2018 (Project Identification Code: 20181010).

### 4.2. Fish and Sampling

The embryos, juvenile, and adult individuals of *S. schlegelii* used in this study were obtained from a commercial hatchery located in Huangdao District of Qingdao (Shan Dong, China). A total of 12 tissues of six 2.5-year-old fish (three males and three females) were randomly sampled, containing the heart, kidney, spleen, liver, brain, gill, pituitary, muscle, intestine, testis, ovary, and ovarian-wall. Fast-twitch fibers and slow-twitch fibers (1.5-year-old fish and 2.5-year-old fish, respectively), and embryo samples at different development stages (1 cell, 32 cells, blastula, gastrula, neural, 8-somites, pre-hatching, pre-fertilization, pre-mating, and post-mating) were collected. All samples were immediately frozen in liquid nitrogen and stored at −80 °C. Tissues from the 70-day-old juvenile slow-twitch muscle fiber domains were prepared for TEM by overnight fixation at 4 °C in 2% glutaraldehyde in 0.1 M phosphate buffer (pH 7.4), and processed according to standard procedures as described in a previous study.

### 4.3. Phylogenetic Analysis of Pax3 and Pax7 Genes

The *Pax3* genes and *Pax7* genes were identified from large yellow croaker (*Larimichthys crocea*), olive flounder (*Paralichthys olivaceus*), Nile tilapia (*Oreochromis niloticus*), amazon molly (*Poecilia formosa*), fugu (*Takifugu rubripes*), tongue Sole (*Cynoglossus semilaevis*), medaka (*Oryzias latipes*), spotted green pufferfish (*Tetraodon nigroviridis*), spotted gar (*Lepisosteus oculatus*), and yellow perch (*Perca flavescens*), based on the National Center for Biotechnology Information (NCBI) or Ensembl database, and the genebank numbers of all genes are available in Table S1. The structure of *SsPax3* and *SsPax7* genes were determined based on the genome and transcriptome data. The schematic diagram was conducted by the online Gene Structure Display Server 2.0 (http://gsds.cbi.pku.edu.cn, accessed on 5 January 2021). The multiple alignment of amino acid sequences was performed by MUSCLE [53]. Phylogenetic trees were constructed using a maximum likelihood algorithm by MEGA 6.06 program with a bootstrap of 1000 replicates. To generate the synteny sketch map of *SsPax3* and *SsPax7* genes and their adjacent genes, the published reference vertebrate included in NCBI database and the genome data of *S. schlegelii* (accession ID CNP0000222) were included.

### 4.4. Protein 3D Structure Predictions of SsPax3 and SsPax7

The 3D structures of two *Pax3* proteins and two *Pax7* proteins were predicted by Phyre2 (http://www.sbg.bio.ic.ac.uk/phyre2/html/page.cgi?id = index, accessed on 11 January 2021) according to the instructions of the website. The figures of protein structure were exported by Chimera software (production version 1.14), San Francisco, CA, USA.

### 4.5. Expression Analysis Using the Available RNA-seq Libraries and Quantitative Real-Time PCR (qRT-PCR)

The transcriptome data of *S. schlegelii* containing 71 RNA-sequence libraries (21 tissues from 2.5-year-old fish and 29 embryonic development stages (https://db.cngb.org/search/sample/?Q=CNP0000222, accessed on 5 January 2021) was utilized to evaluate the expression of *Pax3* and *Pax7*. RNA-sequence reads were mapped to the genome of *S. schlegelii*, and salmon’s gene-expression levels were calculated using default parameters [34]. The expression of target genes was visualized using the Heatmap software package (R 3.5.0 software).

qRT-PCR was performed to examine the expression patterns of *SsPax3* and *SsPax7* in different tissues and injured muscle at different days post-injury. The total RNA of all samples was extracted using Trizol Regant (Invitrogen, California, USA) according to the standard protocol, and then the RNA was immediately stored at −80 °C after removing genomic DNA and protein. Agarose gel electrophoresis and spectrophotometry were adopted to determine the quality and quantity of total RNA. According to the manufacturer’s instructions, 1 μg of total RNA was transcribed into complementary DNA (cDNA) using a reverse transcriptase M-MLV kit (TaKaRa, Dalian, China). Gene-specific primers available in Table 1 were designed to amplify *SsPax3* genes and *SsPax7* genes. The primer pairs were designed by the software Primer Primer v.5.0 [54] shown in Table 1 qRT-PCR was performed by a Light-Cycler 480 real-time PCR system (Roche, Munich, Germany) with 20 μL of reaction volume, which contained 2 μL (5 ng/μL) of cDNA template, 10 μL of 2X SYBR Green qRT-PCR Master Mix (US Everbright Inc., Suzhou, China), 7.2 μL of nuclease-free water, and 0.4 μL of each primer. The following PCR profile was used: pre-incubated at 95 °C for 5 min and 45 cycles at 95 °C for 15 s and 60 °C for 45 s. The reaction of each sample was set by three replicates, and *Eif5a1* was selected as the reference gene. Melting curves were generated after amplification reaction completion to confirm the specificity of the amplicons, and the relative gene-expression levels were calculated using the 2^−ΔΔCt^ comparative Ct method [55]. The statistical analysis of tissue expression of the four genes was carried out using a one-way ANOVA method followed Tukey HSD (subset for alpha = 0.05). Differences in the relative expression between slow-twitch and fast-twitch, and between control and injured were tested by an independent-samples *t*-test by SPSS 2.0 (IBM, Armonk, USA), and each experiment was performed with triplicates. Significance was set at *p* value < 0.05.

### 4.6. Muscle Regeneration Experiment

Thirty juvenile fish of *S. schlegelii* with an average body weight of 41.3 ± 8.8 g were used for the muscle regeneration experiment. A total of five sampling points were set up, which were at 0, 2, 4, 8, and 16 days post-injury, and the number of samples at each time point was six. A sterile surgical blade injured the left trunk, dorsal fin back, and lateral line above the skeletal muscle. The undamaged muscle of the corresponding position on the other side was used as the control group. During the experiment, all the fish survived without infection. Part of the injured muscle samples were stored in 4% paraformaldehyde (PFA) for 24 h at 4 °C, dehydrated with 30%, 50%, 75%, 95% methanol, with each concentration lasting for 2 h, and then stored in 100% methanol. The dehydrated tissues were used for tissue section preparation, embedded in paraffin, followed by being sectioned at a thickness of 4–5 μm. Routine hematoxylin–eosin (H–E) staining was used to observe the morphology of muscle fibers under a Nikon Eclipse Ti-U microscope (Nikon, Tokyo, Japan). The other samples were quickly frozen in liquid nitrogen and then stored at −80 °C. Prior to sampling, MS-222 (20 mg/L) was used for anesthesia.

### 4.7. In Situ Hybridization (ISH)

The slow-twitch muscle fibers of juvenile fish (41.3 ± 8.8 g) were used for the ISH experiment. The ISH probes of two *SsPax3* genes and two *SsPax7* genes were synthesized using a Digoxigenin (DIG)-labeled RNA labelling kit (Roche, Mannheim, Germany). ISH for muscle tissues and WISH for different embryo stages were performed as previously described [56]. The cross-sections of muscle fibers were observed and photographed under a Nikon Eclipse Ti-U microscope, and the stained embryos were placed in glycerol and viewed under a stereomicroscope.

## 5. Conclusions

We characterized two duplicated *Pax3* genes and two duplicated *Pax7* genes in a marine teleost, *S. schlegelii*, which showed indeterminate growth. The embryonic origin tracing of muscle satellite cells revealed their heterozygosity in *S. schlegelii*. To date, we have traced the earliest embryonic origin of muscle satellite cells to the multi-cell stage. Furthermore, during the regenerating process after injury, *SsPax3a* and two *SsPax7* genes were significantly up-regulated, while *SsPax3b* stayed stable, indicating that the satellite cells labeled by *SsPax3b* were not involved in muscle repair. Our study deepens the understanding of the molecular mechanisms of indeterminate muscle growth and regeneration in large fish species.

## Figures and Tables

**Table 1:** Primers used in this study.

Primer Name	Sequence
SsPax3a-qRT-PCR-Fw	GGGTCGTATTCTCATGGTTATC
SsPax3a-qRT-PCR-Rv	GGTTGCAGTGGTTTCACTA
SsPax3b-qRT-PCR-Fw	CCATTCACCAGGACACATT
SsPax3b-qRT-PCR-Rv	GCTGCACCAACCTCATTA
SsPax7a-qRT-PCR-Fw	TGTACTTTGACCTTGCTGTT
SsPax7a-qRT-PCR-Rv	CTCATCATCCAGTCGTGTTT
SsPax7b-qRT-PCR-Fw	TCAGCTGTACTGGGACTG
SsPax7b-qRT-PCR-Rv	ACACACACACACACACAC
SsEif5a1-qRT-PCR-Fw	CTTTGCTCTGGTTCCTGAGTGG
SsEif5a1-qRT-PCR-Rv	AGCTTTGACATGCTGGGGTG
SsPax3a-ISH-Fw	ATTTAGGTGACACTATAGAAGAGGACAGAAAGACAAGAGGTACAG
SsPax3a-ISH-Rv	TAATACGACTCACTATAGGGAGACCATTAAGCGGCTATTGTAAAC
SsPax3b-ISH-Fw	ATTTAGGTGACACTATACGCGGATCCAGGCATCACTTCAAACATGG
SsPax3b-ISH-Rv	TAATACGACTCACTATAGCGAGCTCCCTCATTTGTCTCCACCTT
SsPax7a-ISH-Fw	ATTTAGGTGACACTATAGAAGAGGGACAGGCCTACTAAAG
SsPax7a-ISH-Rv	TAATACGACTCACTATAGGGAGACCATGACAAACAGGAAC
SsPax7b-ISH-Fw	ATTTAGGTGACACTATAGAAGAGACATACAGCACAACCAG
SsPax7b-ISH-Rv	TAATACGACTCACTATAGGGAGACAGCTGCATCATTCAATC

## Supplementary Materials

The following are available online at https://www.mdpi.com/article/10.3390/ijms22073769/s1, Table S1: The genebank numbers of all genes.

## Abbreviations

*Pax3*	Paired box protein *Pax-3*
*Pax3a*	Paired box protein *Pax-3a*
*Pax3b*	Paired box protein *Pax-3b*
*Pax7*	Paired box protein *Pax-7*
*Pax7a*	Paired box protein *Pax-7a*
*Pax7b*	Paired box protein *Pax-7b*
*MyoD*	Myoblast determination protein
*Myf5*	Myogenic factor 5

## References

[1] Sandow A. (1970). Skeletal Muscle. Annu. Rev. Physiol..

[2] Choi Y.M., Suh Y., Shin S., Lee K. (2014). Skeletal Muscle Characterization of Japanese Quail Line Selectively Bred for Lower Body Weight as an Avian Model of Delayed Muscle Growth with Hypoplasia. PLoS ONE.

[3] Biga P.R., Goetz F.W. (2006). Zebrafish and giant danio as models for muscle growth: Determinate vs. indeterminate growth as determined by morphometric analysis. Am. J. Physiol. Integr. Comp. Physiol..

[4] Wang Y.X., Rudnicki M.A. (2011). Satellite cells, the engines of muscle repair. Nat. Rev. Mol. Cell Biol..

[5] Ruparelia A.A., Ratnayake D., Currie P.D. (2019). Stem cells in skeletal muscle growth and regeneration in amniotes and teleosts: Emerging themes. Wiley Interdiscip. Rev. Dev. Biol..

[6] Mauro A. (1961). Satellite Cell of Skeletal Muscle Fibers. J. Cell Biol..

[7] Kuang S., Chargé S.B., Seale P., Huh M., Rudnicki M.A. (2006). Distinct roles for Pax7 and Pax3 in adult regenerative myogenesis. J. Cell Biol..

[8] Buckingham M. (2007). Skeletal muscle progenitor cells and the role of Pax genes. Comptes Rendus Biol..

[9] Buckingham M., Relaix F. (2007). The Role ofPaxGenes in the Development of Tissues and Organs: Pax3 and Pax7 Regulate Muscle Progenitor Cell Functions. Annu. Rev. Cell Dev. Biol..

[10] Yang Q., Zhang K., Jie M., Xiangbing B., Jun H. (2016). PAX3^+^ skeletal muscle satellite cells retain long-term self-renewal and proliferation. Muscle Nerve.

[11] Kassar-Duchossoy L., Giacone E., Gayraud-Morel B., Jory A., Gomès D., Tajbakhsh S. (2005). Pax3/Pax7 mark a novel population of primitive myogenic cells during development. Genes Dev..

[12] Lang D., Powell S.K., Plummer R.S., Young K.P., Ruggeri B.A. (2007). PAX genes: Roles in development, pathophysiology, and cancer. Biochem. Pharmacol..

[13] Gruss P., Walther C. (1992). Pax in development. Cell.

[14] Paixão-Côrtes V.R., Salzano F.M., Bortolini M.C. (2015). Origins and evolvability of the PAX family. Semin. Cell Dev. Biol..

[15] Tremblay P., Dietrich S., Mericskay M., Schubert F.R., Li Z., Paulin D. (1998). A Crucial Role for Pax3 in the Development of the Hypaxial Musculature and the Long-Range Migration of Muscle Precursors. Dev. Biol..

[16] Schubert F.R., Tremblay P., Mansouri A., Faisst A.M., Kammandel B., Lumsden A., Gruss P., Dietrich S. (2001). Early mesodermal phenotypes in splotch suggest a role for Pax3 in the formation of epithelial somites. Dev. Dyn. Off. Publ. Am. Assoc. Anat..

[17] Crist C.G., Montarras D., Pallafacchina G., Rocancourt D., Cumano A., Conway S.J., Buckingham M. (2009). Muscle stem cell behavior is modified by microRNA-27 regulation of Pax3 expression. Proc. Natl. Acad. Sci. USA.

[18] Mansouri A., Gruss P. (1998). Pax3 and Pax7 are expressed in commissural neurons and restrict ventral neuronal identity in the spinal cord. Mech. Dev..

[19] Le Grand F., A Rudnicki M. (2007). Skeletal muscle satellite cells and adult myogenesis. Curr. Opin. Cell Biol..

[20] Maroto M., Reshef R., Münsterberg A.E., Koester S., Goulding M., Lassar A.B. (1997). Ectopic Pax-3 activates MyoD and Myf-5 ex-pression in embryonic mesoderm and neural tissue. Cell.

[21] Bajard L., Relaix F., Lagha M., Rocancourt D., Daubas P., Buckingham M.E. (2006). A novel genetic hierarchy functions during hypaxial myogenesis: Pax3 directly activates Myf5 in muscle progenitor cells in the limb. Genes Dev..

[22] De Morree A., Klein J.D.D., Gan Q., Farup J., Urtasun A., Kanugovi A., Bilen B., Van Velthoven C.T.J., Quarta M., Rando T.A. (2019). Alternative polyadenylation of Pax3 controls muscle stem cell fate and muscle function. Science.

[23] Der Vartanian A., Quétin M., Michineau S., Auradé F., Hayashi S., Dubois C., Rocancourt D., Drayton-Libotte B., Szegedi A., Buckingham M. (2019). PAX3 Confers Functional Heterogeneity in Skeletal Muscle Stem Cell Responses to Environmental Stress. Cell Stem Cell.

[24] Relaix F., Montarras D., Zaffran S., Gayraud-Morel B., Rocancourt D., Tajbakhsh S., Mansouri A., Cumano A., Buckingham M. (2005). Pax3 and Pax7 have distinct and overlapping functions in adult muscle progenitor cells. J. Cell Biol..

[25] Seale P., Sabourin L.A., Girgis-Gabardo A., Mansouri A., Gruss P., Rudnicki M.A. (2000). Pax7 Is Required for the Specification of Myogenic Satellite Cells. Cell.

[26] Oustanina S., Hause G., Braun T. (2004). Pax7 directs postnatal renewal and propagation of myogenic satellite cells but not their specification. EMBO J..

[27] Berberoglu M.A., Gallagher T.L., Morrow Z.T., Talbot J.C., Hromowyk K.J., Tenente I.M., Langenau D.M., Amacher S.L. (2017). Satellite-like cells contribute to pax7-dependent skeletal muscle repair in adult zebrafish. Dev. Biol..

[28] Noden D.M. (1991). Cell movements and control of patterned tissue assembly during craniofacial development. J. Craniofacial Genet. Dev. Boil..

[29] Trainor A.P., Tan S.S., Tam P.P. (1994). Cranial paraxial mesoderm: Regionalisation of cell fate and impact on craniofacial development in mouse embryos. Development.

[30] Gros J., Manceau M., Thomé V., Marcelle C. (2005). A common somitic origin for embryonic muscle progenitors and satellite cells. Nature.

[31] Ordahl C.P., Williams B.A., Denetclaw W. (1999). Determination and morphogenesis in myogenic progenitor cells: An experimental embryological approach. Curr. Top. Dev. Biol..

[32] Hollway G.E., Bryson-Richardson R.J., Berger S., Cole N.J., Hall T.E., Currie P.D. (2007). Whole-Somite Rotation Generates Muscle Progenitor Cell Compartments in the Developing Zebrafish Embryo. Dev. Cell.

[33] Lepper C., Conway S.J., Fan C.-M. (2009). Adult satellite cells and embryonic muscle progenitors have distinct genetic requirements. Nat. Cell Biol..

[34] He Y., Chang Y., Bao L., Yu M., Li R., Niu J., Fan G., Song W., Seim I., Qin Y. (2019). A chromosome-level genome of black rockfish, Sebastes schlegelii, provides insights into the evolution of live birth. Mol. Ecol. Resour..

[35] Gibson M.C., Schultz E. (1982). The distribution of satellite cells and their relationship to specific fiber types in soleus and extensor digitorum longus muscles. Anat. Rec. Adv. Integr. Anat. Evol. Biol..

[36] Holland L.Z., Schubert M., Kozmik Z., Holland N.D. (1999). AmphiPax3/7, an amphioxus paired box gene: Insights into chordate myogenesis, neurogenesis, and the possible evolutionary precursor of definitive vertebrate neural crest. Evol. Dev..

[37] Jiao S., Tan X., Wang Q., Li M., Du S.J. (2015). The olive flounder (Paralichthys olivaceus) Pax3 homologues are highly conserved, encode multiple isoforms and show unique expression patterns. Comp. Biochem. Physiol. Part B Biochem. Mol. Biol..

[38] Akolkar D.B., Asaduzzaman M., Kinoshita S., Asakawa S., Watabe S. (2016). Characterization of Pax3 and Pax7 genes and their expression patterns during different development and growth stages of Japanese pufferfish Takifugu rubripes. Gene.

[39] Sui Y., Tan X., Jun S.Y., Feng M., Jiao S. (2015). The duplicated paired box protein 7 (pax7) genes differentially transcribed during Japanese flounder (*Paralichthys olivaceus*) embryogenesis. Comp. Biochem. Physiol. Part B Biochem. Mol. Biol..

[40] Treisman J., Harris E., Desplan C. (1991). The paired box encodes a second DNA-binding domain in the paired homeo domain protein. Genes Dev..

[41] Goulding M.D., Chalepakis G., Deutsch U., Erselius J.R., Gruss P. (1991). Pax-3, a novel murine DNA binding protein expressed during early neurogenesis. EMBO J..

[42] Goulding M., Lumsden A., Paquette A.J. (1994). Regulation of Pax-3 expression in the dermomyotome and its role in muscle devel-opment. Development.

[43] Jostes B., Walther C., Gruss P. (1990). The murine paired box gene, Pax7, is expressed specifically during the development of the nervous and muscular system. Mech. Dev..

[44] Seo H.-C., Sætre B.O., Håvik B., Ellingsen S., Fjose A. (1998). The zebrafish Pax3 and Pax7 homologues are highly conserved, encode multiple isoforms and show dynamic segment-like expression in the developing brain. Mech. Dev..

[45] Neal A., Boldrin L., Morgan J.E. (2012). The Satellite Cell in Male and Female, Developing and Adult Mouse Muscle: Distinct Stem Cells for Growth and Regeneration. PLoS ONE.

[46] Thompson J.A., Zembrzycki A., Mansouri A., Ziman M. (2008). Pax7 is requisite for maintenance of a subpopulation of superior col-licular neurons and shows a diverging expression pattern to Pax3 during superior collicular development. BMC Dev. Biol..

[47] Maczkowiak F., Mateos S., Wang E., Roche D., Harland R., Monsoro-Burq A.H. (2010). The Pax3 and Pax7 paralogs cooperate in neural and neural crest patterning using distinct molecular mechanisms, in Xenopus laevis embryos. Dev. Biol..

[48] Christ B., Ordahl C.P. (1995). Early stages of chick somite development. Beiträge Ref. Anat. Entwickelungsgeschichte.

[49] Prisk V., Huard J. (2003). Muscle injuries and repair: The role of prostaglandins and inflammation. Histol. Histopathol..

[50] Tierney M.T., Stec M.J., Rulands S., Simons B.D., Sacco A. (2018). Muscle Stem Cells Exhibit Distinct Clonal Dynamics in Response to Tissue Repair and Homeostatic Aging. Cell Stem Cell.

[51] Chargé S.B.P., Rudnicki M.A. (2004). Cellular and Molecular Regulation of Muscle Regeneration. Physiol. Rev..

[52] Tian Z.-L., Jiang S.-K., Zhang M., Wang M., Li J.-Y., Zhao R., Wang L.-L., Li S.-S., Liu M., Zhang M.-Z. (2015). Detection of satellite cells during skeletal muscle wound healing in rats: Time-dependent expressions of Pax7 and MyoD in relation to wound age. Int. J. Leg. Med..

[53] Tamura K., Stecher G., Peterson D., Filipski A., Kumar S. (2001). Molecular Evolutionary Genetics Analysis Version 6.0. Mol. Biol. Evol..

[54] Lalitha S. (2000). Primer premier 5. Biotech. Softw. Internet Rep. Comput. Softw. J. Sci..

[55] Pfaffl M.W. (2001). A new mathematical model for relative quantification in real-time RT-PCR. Nucleic Acids Res..

[56] Wang B., Du X., Wang H., Jin C., Gao C., Liu J., Zhang Q. (2019). Comparative studies on duplicated tdrd7 paralogs in teleosts: Mo-lecular evolution caused neo-functionalization. Comp. Biochem. Physiol. Part D Genom. Proteom..

