# Supplementary material for: Pax3 and Pax7 Exhibit Distinct and Overlapping Functions in Marking Muscle Satellite Cells and Muscle Repair in a Marine Teleost, Sebastes schlegelii
Source: Int J Mol Sci. 2021 Apr 5;22(7):3769. doi: 10.3390/ijms22073769 (PMC8038590; doi:10.3390/ijms22073769)
Supplement: Supplementary file 1 [file ijms-22-03769-s001.pdf]

Supplementary Materials:

Table S1: The genebank numbers of all genes.

Species	Gene	Accession number
Homo sapiens	*Pax3*	ENSG00000135903
Gallus gallus	*Pax3*	ENSGALP00000008414
Xenopus laevis	*Pax3*	XP_018120039.1
Mus musculus	*Pax3*	ENSMUSP00000084320
Sebastes schlegelii	*Pax3a*	*S.schlegelii*_GLEAN_10013367
Sebastes schlegelii	*Pax3b*	*S.schlegelii*_GLEAN_10013092
Oreochromis niloticus	*Pax3a*	ENSONIG00000010028
Oreochromis niloticus	*Pax3b*	ENSONIG00000014632
Takifugu rubripes	*Pax3a*	ENSTRUG00000013444
Takifugu rubripes	*Pax3b*	ENSTRUG00000009198
Poecilia formosa	*Pax3a*	ENSPFOG00000007769
Poecilia formosa	*Pax3b*	ENSPFOG00000014283
Larimichthys crocea	*Pax3a*	XP_010729472.2
Larimichthys crocea	*Pax3b*	XP_019111823.1
Cynoglossus semilaevis	*Pax3a*	XP_008332152.1
Cynoglossus semilaevis	*Pax3b*	ENSCSEG00000005432
Oryzias latipes	*Pax3a*	ENSORLG00000015932
Oryzias latipes	*Pax3b*	ENSORLG00000009031
Paralichthys olivaceus	*Pax3a*	XP_019968168
Paralichthys olivaceus	*Pax3b*	XP_019950891.1
Tetraodon nigroviridis	*Pax3a*	ENSTNIG00000015279
Tetraodon nigroviridis	*Pax3b*	ENSTNIG00000014313
Lepisosteus oculatus	*Pax3b*	ENSLOCG00000004189
Perca flavescens	*Pax3a*	XP_028448440.1
Perca flavescens	*Pax3b*	XP_028428285.1
Ascaris lumbricoides	*Ag1*	ACJ03764.1
Homo sapiens	*Pax7*	CAA65522.1
Gallus gallus	*Pax7*	NP_990396.1
Xenopus laevis	*Pax7*	NP_001088995.1
Mus musculus	*Pax7*	AAG16663.3
Sebastes schlegelii	*Pax7a*	*S.schlegelii*_GLEAN_10012719
Sebastes schlegelii	*Pax7b*	*S.schlegelii*_GLEAN_10017555
Oreochromis niloticus	*Pax7a*	XP_025763203.1
Oreochromis niloticus	*Pax7b*	ENSONIG00000001596
Takifugu rubripes	*Pax7a*	XP_029682981.1
Takifugu rubripes	*Pax7b*	ENSTRUG00000015245.1
Poecilia formosa	*Pax7a*	XP_007549743.1
Poecilia formosa	*Pax7b*	XP_016518023.1
Larimichthys crocea	*Pax7a*	XP_010729647.3
Larimichthys crocea	*Pax7b*	XP_019111823.1
Cynoglossus semilaevis	*Pax7a*	XP_008317794.2
Cynoglossus semilaevis	*Pax7b*	XP_024915390.1
Oryzias latipes	*Pax7a*	NP_001292564.1
Oryzias latipes	*Pax7b*	ENSORLG00000004269
Paralichthys olivaceus	*Pax7a*	ALA23008.1
Paralichthys olivaceus	*Pax7b*	ALA23014.1
Gasterosteus aculeatus	*Pax7a*	ENSGACG00000012890

Gasterosteus aculeatus	*Pax7b*	ENSGACT00000017105
Tetraodon nigroviridis	*Pax7a*	ENSLOCG00000003864
Lepisosteus oculatus	*Pax7a*	XP_006642005.1
Lepisosteus oculatus	*Pax7b*	ENSLOCT00000004685
Perca flavescens	*Pax7a*	XP_028430685.1
Perca flavescens	*Pax7b*	XP_028437964.1
Danio rerio	*Pax7a*	AAI63523.1
Danio rerio	*Pax7b*	ACN88553.1
